# From PCOS to PMOS: Change of name or change of paradigm? What the general practitioner needs to know?

**DOI:** 10.61622/rbgo/2026rbgoedt4

**Published:** 2026-10-13

**Affiliations:** 1 Universidade de São Paulo Faculdade de Medicina São Paulo SP Brazil Faculdade de Medicina, Universidade de São Paulo, São Paulo, SP, Brazil; 2 Universidade de São Paulo Faculdade de Medicina Ribeirão Preto SP Brazil Faculdade de Medicina, Universidade de São Paulo, Ribeirão Preto, SP, Brazil; 3 Universidade Federal de Mato Grosso Cuiabá MT Brazil Universidade Federal de Mato Grosso, Cuiabá, MT, Brazil

**Keywords:** Polycystic ovary syndrome, Metabolic syndrome, Polyendocrine metabolic ovarian syndrome

Polycystic Ovary Syndrome (PCOS) is recognized as one of the most common and complex conditions in clinical practice, affecting approximately one in eight women worldwide. Recently, an international initiative proposed changing its name to Polyendocrine Metabolic Ovarian Syndrome (PMOS).^(1)^ For the practicing physician, the question arises: what does this change in practice?

The short and fundamental answer is: there is no change in the established pathophysiology, diagnosis, or treatment. The proposed new nomenclature aims only to better reflect the multisystemic nature of the disorder, but the scientific basis supporting patient care remains the same.^(2)^

## The diagnosis: The foundation remains rotterdam

Despite the discussion regarding the name,^(1,3)^ diagnostic criteria continue to follow the recommendations of the 2023 International Guideline.^(2)^ Diagnosis is still based on the presence of at least two of the three Rotterdam criteria:

1. Ovulatory dysfunction (irregular cycles).
2. Hyperandrogenism (clinical, such as hirsutism and acne, or biochemical).
3. Polycystic ovary morphology on ultrasound or, alternatively, elevated levels of Anti-Müllerian Hormone (AMH).

It is important to clarify the precise role of AMH in this updated framework. According to the 2023 guideline,^(2)^ AMH serves as an alternative to ultrasound for defining polycystic ovarian morphology (PCOM) in adults, particularly when high-quality ultrasound is unavailable or unacceptable to the patient.^(1,2)^ However, AMH should not be considered a direct equivalent to ultrasound in all circumstances. The choice between AMH and ultrasound should be guided by patient factors (e.g., age, BMI) and access, and both tests should not be used together, as this would lead to overdiagnosis. Furthermore, AMH is not recommended for use in adolescents due to poor specificity and the high prevalence of physiological multifollicular ovaries in this age group. Therefore, while AMH is a valuable diagnostic tool, its role remains complementary and conditional, not a wholesale replacement for ultrasound.^(2)^ In reality, both ovarian ultrasound and AMH measurements are subject to interpretive pitfalls in adolescents, owing to physiological characteristics of this age group—namely, the presence of multifollicular ovaries and naturally higher AMH levels compared to adult women. Importantly, neither the AE-PCOS Society nor the Endocrine Society mandates these tests as obligatory for the diagnosis of PCOS/PMOS in adolescents. Our group has consistently maintained a critical stance regarding the routine use of both examinations, even in adult women, given their potential for overdiagnosis and misinterpretation when not applied judiciously.

For adolescents, however, a different approach is recommended. In this age group, ultrasound is not indicated for diagnosis, because multifollicular ovaries are common during puberty and do not necessarily reflect PCOM. Therefore, if an adolescent presents with only irregular cycles OR hyperandrogenism (without fulfilling both criteria), she should be considered "at risk" for PCOS/PMOS and reassessed later with re-evaluation annually or as clinically indicated, rather than rigidly waiting up to 8 years post-menarche, because the clinical picture may become evident much earlier and early intervention can modify the disease course.^(1,2)^ This cautious approach helps avoid overdiagnosis and unnecessary anxiety in young patients.

It is vital to clarify that the term "polycystic" has always been a misnomer, as what is observed are not cysts, but rather follicles that have arrested their development. The shift to PMOS attempts to correct this historical error and reduce the stigma and anxiety of patients who fear "cysts" or premature ovarian insuficiency.^(1)^

## Pathophysiology and treatment: The focus on insulin resistance

The essence of the syndrome remains linked to the dysregulation of the hypothalamus-pituitary-ovary axis and, frequently, to insulin resistance (IR). Regardless of the name, lifestyle modification remains the pillar of treatment. In this context, new evidence brings practical tools for clinical management. A recent meta-analysis published in Nutrients^(4,5)^ evaluated Time-Restricted Eating (TRE) in women with PCOS/PMOS. Although promising, the data should be interpreted with caution; the studies are heterogeneous, short-term, and the metabolic advantages over conventional caloric restriction remain debatable. Thus, while TRE may be an option for some patients, it should not be presented as a clearly superior strategy. Beyond lifestyle interventions, pharmacotherapy has evolved significantly. Glucagon-like peptide-1 (GLP-1) receptor agonists (e.g., semaglutide, liraglutide and tirzepatide) have emerged as powerful adjuncts in managing PCOS/PMOS, particularly for patients with obesity and significant insulin resistance. These agents promote substantial weight loss and directly improve glycemic control and IR, often with greater efficacy than metformin alone in selected cases.^(6)^ Although not yet first-line therapy, their use is increasingly integrated into clinical practice, offering a valuable perspective for patients who do not achieve adequate metabolic control with lifestyle changes alone.

## Criticisms of the new nomenclature

Although the term PMOS seeks to highlight the metabolic side,^(1)^ it is not without flaws. The term "Polyendocrine" may be technically imprecise, as the syndrome is not a failure of multiple independent glands (as in autoimmune syndromes), but rather a functional dysregulation.^(7)^ Furthermore, labeling the syndrome primarily as "Metabolic" may stigmatize lean patients who possess normal metabolic profiles. Additionally, although the discussion of "Ovary" from the name was suggested following a global consultation of experts,^(1)^ we understand that the term "Ovary" should never be entirely omitted. The clinical manifestations that prompt patients to seek medical attention are overwhelmingly reproductive in nature namely, menstrual irregularities, infertility, and hyperandrogenism (hirsutism, acne, and androgenic alopecia). Removing "Ovary" from the nomenclature risks detaching the syndrome from the very symptoms that define the patient's experience and drive their initial consultation. Any new name must retain a clear link to ovarian function to remain clinically meaningful and patient-centered.

## Conclusion for clinical practice

The transition from PCOS to PMOS is an attempt to modernize medical communication and align the disease's name with what it truly represents in a woman's body. However, for the general practitioner, management remains focused on individualization:

- Treat the patient, not just the name;
- Diagnosis should follow current international guidelines, which are largely based on the Rotterdam or NIH or AE-PCOS criteria while keeping in mind their recognized limitations – particularly the fact that "polycystic" morphology refers to follicular arrest, not true ovarian cysts;
- Encourage sustainable lifestyle changes, considering that strategies such as time-restricted eating (TRE) can be adjunctive tools, though not superior to other evidence-based dietary approaches. In more resistant cases, GLP-1 receptor agonists represent a growing therapeutic option to address the core metabolic dysfunction;
- Science progresses in how we name diseases, but the commitment to treatment based on evidence and known pathophysiology remains unchanged.

## Data Availability

The research data are described in the article presented.
